# Preparation, carbonic anhydrase enzyme inhibition and antioxidant activity of novel 7-amino-3,4-dihydroquinolin-2(1H)-one derivatives incorporating mono or dipeptide moiety

**DOI:** 10.1080/14756366.2020.1751620

**Published:** 2020-04-16

**Authors:** Hasan Küçükbay, Zeynep Gönül, F. Zehra Küçükbay, Andrea Angeli, Gianluca Bartolucci, Claudiu T. Supuran

**Affiliations:** a Department of Chemistry, Faculty of Arts and Sciences, İnönü University, Malatya, Turkey; b Department of Basic Pharmaceutical Sciences, Faculty of Pharmacy, İnönü University, Malatya, Turkey; c Dipartimento Neurofarba, Sezione Di Scienze Farmaceutiche E Nutraceutiche e Laboratorio Di Chimica Bioinorganica, Università Degli Studi Di Firenze, Florence, Italy

**Keywords:** Dipeptide, dihydroquinolinone derivatives, carbonic anhydrase, antioxidant

## Abstract

New dipeptide–dihydroquinolinone derivatives were successfully synthesised by benzotriazole mediated nucleophilic acyl substitution reaction and their structures were elucidated by spectroscopic and analytic techniques. The carbonic anhydrase (CA, EC 4.2.1.1) inhibitory activity of the new compounds was determined against four human (h) isoforms, hCA I, hCA II, hCA IX and hCA XII. While all compounds showed moderate to good *in vitro* CA inhibitory properties against hCA IX and hCA XII with inhibition constants in the micromolar level (37.7–86.8 and 2.0–8.6 µM, respectively), they did not show inhibitory activity against hCA I and hCA II up to 100 µM concentration. The antioxidant capacity of the peptide–dihydroquinolinone conjugates was determined using 1,1-diphenyl-2-picrylhydrazyl (DPPH) radical scavenging method. Most of the synthesised compounds showed low antioxidant activities compared to the control antioxidant compounds BHA and α-tocopherol.

## 1. Introduction

2-Quinolinones derivatives constitute a privilege class of heterocyclic compounds for their wide range of important biological properties such as such as antibacterial^1^^,^^2^, antimalarial^3^, antitumor^4^, carbonic anhydrase inhibitor^5^^,^^6^, antioxidant, anti-tuberculosis^7^, antiparasitic^8^ and anti-hepatit C and B viruses activity^9^. For example, the 3,4-dihydro-2-quinolinone structure is found in a number of biologically active and FDA approved medicine such as cilostazol, carteolol and aripiprazole. Compounds containing the 3,4-dihydro-2(1H)-quinolone moiety also exhibit a variety of activities in both the peripheral and central tissues, which includes phosphodiesterase inhibition, blocking of β-adrenergic receptors, antagonism of vasopressin receptors and interaction with serotonin and dopamine receptors^10^. Since heterocyclic compounds containing peptide have been of particular interest because their biocompatibility of the peptide parts play a crucial role in transporting into mammalian tissue of these type drug candidates^11^. The carbonic anhydrase enzymes play a role in many physiological events, such as acid base balance, regulation of cardiovascular tone, digestion, ion exchange between cell sections and providing the necessary bicarbonate for different enzymatic reactions^12–14^. The emergence of possible relationships between carbonic anhydrase enzyme and cancer in recent years has increased the interest in carbonic anhydrase enzyme inhibitors^15^^,^^16^.

Encouraged by the above literature information and our interest in the biological and chemical properties of such compounds, synthesis and carbonic anhydrase and antioxidant properties of mono and dipeptide containing dihydroquinolinone derivatives have been studied.

## 2. Material and methods

### 2.1. Chemistry

The starting materials and reagents used in the reactions were supplied commercially by Across, Aldrich or Merck Chemical Co. ^1^H NMR (400.13 MHz) and ^13^C NMR (100.62 MHz) spectra were obtained using Bruker Advance 400 Ultra shield high performance digital FT NMR spectrometer. Infra-red spectra were recorded with ATR equipment in the range 4000–200 cm^−1^ on a Perkin-Elmer FT-IR spectrophotometer. Elemental analyses were performed by LECO CHNS-932 elemental analyser. Melting points were recorded using an electrothermal-9200 melting point apparatus and are uncorrected. Positive or negative-ion electrospray ionization (ESI) mass spectra were recorded on a double-focusing Finnigan MAT 95 instrument with BE geometry. All microwave-assisted reactions were carried out in a microwave oven system manufactured by Milestone (Milestone Start S Microwave Labstation for Synthesis). Benzotriazole derivatives of *N*-protected amino acids (I–V)^3^^,^^17^^,^^18^ and dipeptide (VI)^19^^,^^20^ were prepared according to literature procedure.

### 2.2. General procedure for the synthesis of mono or dipeptide–dihydroquinolinone conjugates, 1–6

A mixture of equivalent amounts of the appropriate N-protected aminoacylbenzotriazole and 7-amino-3,4-dihydroquinolin-2(1H)-one was subjected to microwave irradiation (100 W, 70 °C) in anhydrous THF (5 ml) for 30 min. On completion of the reaction followed by TLC, all volatiles were removed by rotavapor and the obtained crude product was crystallised from methanol.

#### 2.2.1. *Tert-butyl (R)-(1-oxo-1-((2-oxo-1,2,3,4-tetrahydroquinolin-7-yl)amino)propan-2-yl)carbamate,* 1


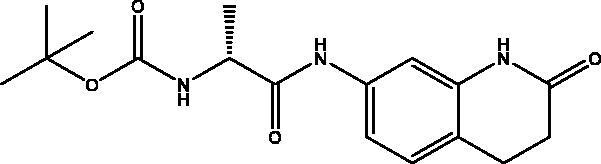


Cream solid (77%); mp 197–198 °C; ^1^H NMR (DMSO-d_6_, 400 MHz), *δ* 10.17 (s, 1H, N*H_lactam_*), 9.91 (s, 1H,NH), 7.28 (d, 1H, N–H, *J* = 4 Hz), 7.17–7.09 (m, 3 H, Ar–*H*) , 4.16–4.13 (m, 1H, C*H*NH), 2.86 (t, 2H, COC*H*_2_, *J* = 8 Hz), 2.47 (t, 2H, COCH_2_C*H*_2_, *J* = 8 Hz), 1.44 (s, 9H, (OCH(*C*H_3_)_3_), 1.29 (d, 3H, CHC*H_3_*, *J* = 8 Hz). ^13^C NMR (DMSO-d_6_, 400 MHz), *δ* 171.8 (*C*OCH_2_), 170.8 (*C*OCH), 155.5 (*C*OOCH_2_Ph), 138.93, 138.51, 128.21, 118.82, 113.29, 106.71 (Ar–*C*), 78.5 (O*C*H(CH_3_)_3_), 50.8 (*C*HNH), 31.1 (CO*C*H_2_C*H_2_*), 28.7 (COCH_2_*C*H_2_), 24.8 (OCH(*C*H_3_)_3_), 18.5 (CH*C*H_3_). *_ν_*(C = O)carbamate: 1605 cm^−1^, *_ν_*(C = O)amide: 1672, 1687 cm^−1^, *_ν_*(N–H)amine: 3200, 3344 cm^−1^. Elemental analysis: C_17_H_23_N_3_O_4_ required C, 61.25; H, 6.95; N, 12.60; S, 6.74, found C, 61.02; H, 6.94; N, 12.63. HRMS *m/z* for C_17_H_23_N_3_O_4_ [M + Na]^+^calcd. 356.1586, found 356.2000.

#### 2.2.2. *Tert-butyl (R)-(1-oxo-1-((2-oxo-1,2,3,4-tetrahydroquinolin-7-yl)amino)-3-phenylpropan-2-yl)carbamate,* 2


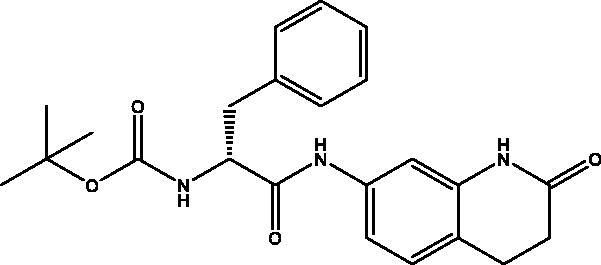


Cream solid (87%); mp 180–181 °C; ^1^H NMR (DMSO-d_6_, 400 MHz), *δ* 10.14 (s, 1H, N*H_lactam_*), 9.99 (s, 1H,NH), 7.33–7.07 (m, 9 H, Ar–*H* + NH) , 4.34–4.29 (m, 1H, C*H*NH), 2.99–2.95 (m, 1H, C*H*_2_Ph), 2.83–2.79 (m, 3H, COC*H*_2_ + C*H*_2_Ph), 2.42 (t, 2H, COCH_2_C*H*_2_), 1.32 (s, 9H, OCH(C*H*_3_)_3_). ^13^C NMR (DMSO-d_6_, 400 MHz), *δ* 171.1 (*C*OCH_2_), 170.8 (*C*OCH), 155.8 (*C*OOC(CH_3_)_3_), 138.9, 138.4, 138.32, 129.7, 128.5, 128.2, 126.6, 1189.0, 113.5, 106.8 (Ar–H),78.7 (O*C*H(CH_3_)_3_), 56.9 (*C*HNH), 37.9 (CH*C*H_2_), 31.1 (CO*C*H_2_C*H_2_*), 28.6 (COCH_2_*C*H_2_), 24.8 (OCH(*C*H_3_)_3_). *_ν_*(C = O)carbamate: 1604 cm^−1^, *_ν_*(C = O)amide: 1623, 1671 cm^−1^, *_ν_*(N–H)amine: 3326 cm^−1^. Elemental analysis: C_23_H_27_N_3_O_4_ required C, 67.46; H, 6.65; N, 10.26, found C, 67.11; H, 6.56; N, 10.30.HRMS *m/z* for C_23_H_27_N_3_O_4_ [M − H]^+^calcd. 408.1923, found 408.2000.

#### 2.2.3. *Tert-butyl* (S)-(4-(methylthio)-1-oxo-1-((2-oxo-1,2,3,4-tetrahydroquinolin-7-yl)amino)butan-2-yl)carbamate, 3


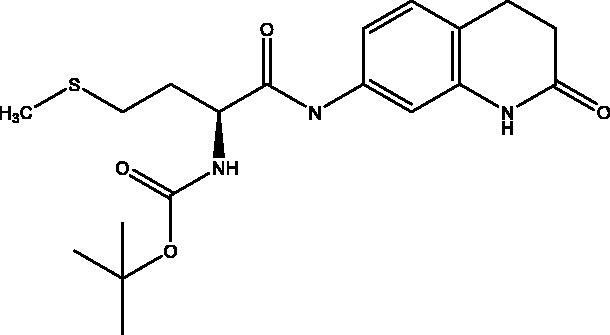


Beige solid (86%); mp 171–172 °C; ^1^H NMR (DMSO-d_6_, 400 MHz), *δ* 10.12 (s, 1H, N*H_lactam_*), 9.93 (s, 1H,NH), 7.23 (d, 1H, N–H, *J* = 4 Hz), 7.13–7.06 (m, 3 H, Ar–*H*) , 4.15–4.10 (m, 1H, C*H*NH), 2.80 (t, 2H, COC*H*_2_, *J* = 8 Hz), 2.48–2.40 (m, 4H, COCH_2_C*H*_2_ + CHC*H_2_*CH_2_S), 2.06 (s, 3H, C*H_3_*), 1.88–1.85 (m, 2H, CHCH_2_C*H_2_*S), 1.38 (s, 9H, OC(C*H*_3_)_3_). ^13^C NMR (DMSO-d_6_, 400 MHz), *δ* 171.2 (*C*OCH_2_), 170.9 (*C*OCH), 156.0 (*C*OOC(CH_3_)_3_), 138.9, 138.3, 128.2, 119.0, 113.5, 106.9 (Ar–H), 78.7 (O*C*H(CH_3_)_3_), 65.4 (*C*HNH), 54.8 (CO*C*H_2_C*H_2_*), 31.1 (CH*C*H_2_CH_2_S), 30.2 (COCH_2_*C*H_2_), 28.6 (S*C*H_3_), 24.8 (CHCH_2_*C*H_2_S), 15.1 (OCH(*C*H_3_)_3_). *_ν_*(C = O)carbamate: 1613 cm^−1^, *_ν_*(C = O)amide: 1669, cm^−1^, *_ν_*(N–H)amine: 3224 cm^−1^. Elemental analysis: C_19_H_27_N_3_O_4_ required: C, 57.99; H, 6.92; N, 10.68; S, 8.15, found: C, 57.89; H, 6.86; N, 10.67; S, 8.18. HRMS *m/z* for C_19_H_27_N_3_O_4_S [M − H]^+^ calcd. 392.1644, found 392.1000; [M + Na]^+^ calcd. 416.1620, found 416.3000.

#### 2.2.4. *Benzyl (R)-(1-oxo-1-((2-oxo-1,2,3,4-tetrahydroquinolin-7-yl)amino)-3-(phenylthio)propan-2-yl)carbamate,* 4


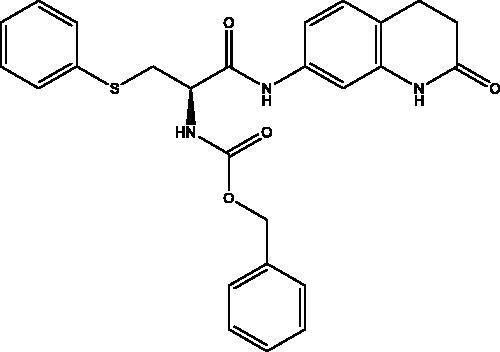


Cream solid (74%); mp 150–151 °C; ^1^H NMR (DMSO-d_6_, 400 MHz), *δ* 10.24 (s, 1H, N*H_lactam_*), 10.20 (s, 1H, NH), 7.88 (d, 1H, N–H, *J* = 8 Hz), 7.45–7.12 (m, 13 H, Ar–*H*) , 5.11 (s, 2H, C*H_2_*Ph), 4.44–4.43 (m, 1H, C*H*NH), 3.42–3.39 (m, 1H, C*H*_2_S), 3.27–3.22 (m, 1H, C*H*_2_S), 2.86 (t, 2H, COC*H*_2_, *J* = 8 Hz), 2.48 (m, 4H, COCH_2_C*H*_2_, *J* = 8 Hz). ^13^C NMR (DMSO-d_6_, 400 MHz), *δ* 170.8 (*C*OCH_2_), 169.0 (*C*OCH), 156.4 (*C*OOCH_2_Ph), 138.9, 138.1, 137.3, 136.3, 129.6, 128.8, 128.8, 128.3, 128.2, 126.4, 119.3, 113.7, 107.1 (Ar–*C*), 66.1 (*C*H_2_Ph), 55.3 (*C*HNH), 35.3 (CH*C*H_2_S), 31.1 (CO*C*H_2_C*H_2_*), 24.8 (COCH_2_*C*H_2_). *_ν_*(C = O)carbamate: 1606 cm^−1^, *_ν_*(C = O)amide: 1659, 1680 cm^−1^, *_ν_*(N–H)amine: 3288 cm^−1^. Elemental analysis: C_26_H_25_N_3_O_4_S required: C, 65.67; H, 5.30; N, 8.84; S, 6.74, found C, 65.39; H, 5.04; N, 8. 71; S, 6.63. HRMS *m/z* for C_26_H_25_N_3_O_4_S [M + H]^+^ calcd. 476.1644, found 476.3000.

#### 2.2.5. *Benzyl (S)-(4-(methylthio)-1-oxo-1-((2-oxo-1,2,3,4-tetrahydroquinolin-7-yl)amino)butan-2-yl)carbamate*, 5


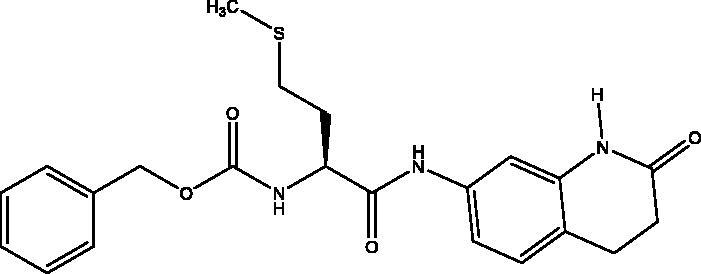


Cream solid (78%); mp 137–138 °C; ^1^H NMR (DMSO-d_6_, 400 MHz), *δ* 10.19 (s, 1H, N*H_lactam_*), 10.08 (s, 1H, NH), 7.70 (d, 1H, N–H, *J* = 8 Hz), 7.51–7.12 (m, 8 H, Ar–*H*) , 5.09 (s, 2H, C*H_2_*Ph), 4.31–4.25 (m, 1H, C*H*NH), 2.86 (t, 2H, COC*H*_2_, *J* = 8 Hz), 2.60–2.39 (m, 4H, CHC*H_2_*CH_2_ + COCH_2_C*H*_2_), 2.10 (s, 3H, CH_3_), 1.99–1.91 (m, 2H, CHCH_2_C*H_2_*SCH_3_). ^13^C NMR (DMSO-d_6_, 400 MHz), *δ* 170.9 (*C*OCH_2_), 170.9 (*C*OCH), 156.6 (*C*OOCH_2_Ph), 138.9, 138.3, 137.4, 128.8, 128.3, 128.2, 119.1, 113.5, 108.5, 106.9 (Ar–*C*), 66.0 (*C*H_2_Ph), 55.2 (*C*HNH), 32.0 (CO*C*H_2_CH_2_), 31.1 (CH*C*H_2_C*H_2_*), 30.2 (COCH_2_*C*H_2_), 24.8 (*C*H_3_), 15.1 (CH_2_*C*H_2_S). *_ν_*(C = O)carbamate: 1604 cm^−1^, *_ν_*(C = O)amide: 1654, 1684 cm^−1^, *_ν_*(N–H)amine: 3283 cm^−1^. Elemental analysis: C_22_H_25_N_3_O_4_S required C, 61.81; H, 5.89; N, 9.83; S, 7.50, found C, 61. 72; H, 5.81; N, 9.62; S, 7.51. HRMS *m/z* for C_22_H_25_N_3_O_4_S [M + H]^+^ calcd. 427.1644, found 428.2000.

#### 2.2.6. *Benzyl ((R)-1-oxo-1-(((S)-1-oxo-1-((2-oxo-1,2,3,4-tetrahydroquinolin-6-yl)amino)-3-phenylpropan-2-yl)amino)-3-phenylpropan-2-yl)carbamate*, 6


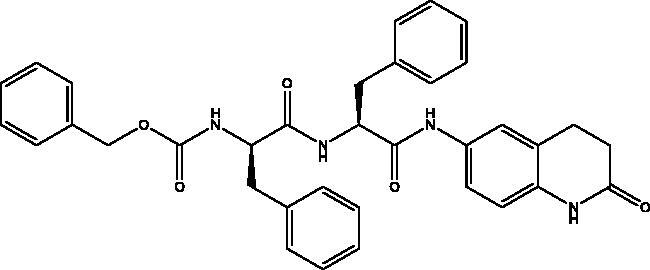


Cream solid (62%); mp 197–198 °C; ^1^H NMR (DMSO-d_6_, 400 MHz), *δ* 10.15 (s, 1H, N*H_lactam_*), 10.06 (s, 1H, NH), 8.30 (d, 1H, N–H, *J* = 8 Hz), 7.47 (d, 1H, N–H, *J* = 8 Hz), 7.33–7.09 (m, 18 H, Ar–*H*) , 4.95 (s, 2H, C*H_2_*Ph), 4.73–4.68 (m, 1H, C*H*NH), 4.32–4.26 (m, 1H, C*H*NH), 3.10–3.06 (m, 1H, C*H*_2_Ph), 2.98–2.91 (m, 2H, C*H*_2_Ph), 2.81 (t, 2H, COC*H*_2_, *J* = 8 Hz), 2.73–2.66 (m, 1H, 1H, C*H*_2_P), 2.42 (t, 2H, COCH_2_C*H*_2_, *J* = 8 Hz). ^13^C NMR (DMSO-d_6_, 400 MHz), *δ* 171.9 (*C*OCH_2_), 170.8, 170.3 (*C*OCH), 156.2 (*C*OOCH_2_Ph), 138.9, 138.4, 138.2, 137.8, 137.5, 129.7, 129.6, 128.8, 128.6, 128.5, 128.2, 128.1, 127.9, 126.9, 126.7, 119.1, 113.5, 106.9 (Ar–*C*), 65.7 (O*C*H_2_Ph), 56.5 (*C*HNH), 55.2 (*C*HNH), 38.3 (CH*C*H_2_), 38.0 (*C*H_2_C*H_2_*), 31.1 (CH*C*H_2_), 24.8 (CH_2_*C*H_2_). *_ν_*(C = O)carbamate: 1608 cm^−1^, *_ν_*(C = O)amide: 1647, 1680 cm^−1^, *_ν_*(N–H)amine: 3282 cm^−1^. Elemental analysis: C35H34N4O5 required C, 71.17; H, 5.80; N, 9.49, found C, 71. 05; H, 5.78; N, 9.21. HRMS *m/z* for C35H34N4O5 [M + H]^+^ calcd. 591.2607, found 593.4000; [M + Na]^+^ calcd. 613.2427, found 613.3000.

### 2.3. Ca inhibition

An Applied Photophysics Stopped-Flow instrument has been used for assaying the CA catalysed CO_2_ hydration activity by using method of Khalifah^21^. Phenol red (at a concentration of 0.2 mM) has been used as indicator, working at the absorbance maximum of 557 nm, with 20 mM HEPES (pH 7.5) as buffer and 20 mM Na_2_SO_4_ (for maintaining constant the ionic strength), following the initial rates of the CA-catalysed CO_2_ hydration reaction for a period of 10–100 s. The CO_2_ concentrations ranged from 1.7 to 17 mM for the determination of the kinetic parameters and inhibition constants. For each inhibitor at least six traces of the initial 5–10% of the reaction have been used for determining the initial velocity. The uncatalysed rates were determined in the same manner and subtracted from the total observed rates. Stock solutions of inhibitor (0.1 mM) were prepared in distilled–deionised water and dilutions up to 0.01 nM were done thereafter with the assay buffer. Inhibitor and enzyme solutions were pre-incubated together for 15 min at room temperature prior to assay, in order to allow for the formation of the E–I complex. The inhibition constants were obtained by non-linear least-square methods using PRISM (www.graphpad.com), and non-linear least squares methods, values representing the mean of at least three different determinations, as described earlier by us^22–27^.

### 2.3. Antioxidant testing

#### 2.3.1. DPPH radical scavenging activity

Antioxidant activity was determined based on the ability of the antioxidants to act as radical scavengers towards the stable free radical, 1,1-diphenyl-2-picrylhydrazyl (DPPH). As detailed by Yang et al^28^, 1 ml of antioxidant solution (solubilised in ethanol) was added to 3 ml of a 0.1 mM ethanolic solution of DPPH. After 30 min at ambient temperature in darkness, absorbance readings were taken at 517 nm. Inhibition (%) was calculated using the equation

[1 – (As−Ao)/Ab]×100

where As was the absorbance reading for samples containing antioxidant, Ao was the absorbance of the antioxidant in pure methanol and Ab corresponded to the absorbance of the DPPH solution.

## 3. Results and discussion

### 3.1. *Synthesis and characterization of the new mono and dipeptide–*dihydroquinolinone *derivatives*

**Scheme 1.**
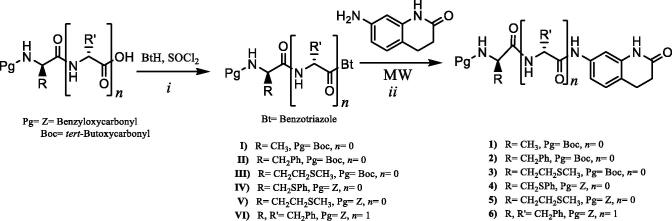
Synthesis pathways of the new dihydroquinolinone conjugates of N-protected amino acids and dipeptide. Conditions and reagents: (i) r.t., 2 h in THF; 70 °C, 30 min in THF.

New N-protected monopeptide and dipeptide–dihydroquinolinone conjugates (1–6) were synthesised by the reaction of 7-amino-3,4-dihydroquinolin-2(1H)-one with *N*-protected aminoacylbenzotriazole under microwave heating at 70 °C for 30 min with good yields of 62–87%. The synthesis of the N-protected mono and dipeptide–dihydroquinolinone conjugates 1–6 is summarised in Scheme 1.

The structures of N-protected mono and dipeptide–dihydroquinolinone conjugates (1–6) were elucidated by ^1^H NMR, ^13^C NMR, IR, mass and elemental analyses. The characteristic *NH* resonances of the lactam moiety of the mono or dipeptide–dihydroquinolinone conjugates in the ^1^H NMR spectra of compounds (1–6) were observed at 10.12–10.24 ppm region as singlet peak. The NH peaks at position 7 of the quinolinone part of the mono and dipeptide–dihydroquinolinone conjugates were observed at 9.91–9.20 ppm region as a singlet peak in the ^1^H NMR spectra. The carbamate NH proton signals for the protected group of the conjugates 1–6 were observed as doublet at 7.23–7.77 ppm, except compound 2 which resonated together with aromatic protons. Other NH peak for compound 6 was observed as doublet at 8.30 ppm. All NH protons were confirmed by deuterium exchange by D_2_O. Carbonyl resonances of the lactam carbonyl, amide carbonyl and carbamate carbonyl for monopeptide–dihydroquinolinone conjugates were observed around 170.8–171.9, 169.0–170.9 and 155.5–156.6 ppm, respectively. Carbonyl resonances of dipeptide–dihydroquinolinone conjugates 6 were appeared at 171.9, 170.8, 170.3 and 156.2 ppm, respectively. All other aliphatic and aromatic protons and carbons for mono and dipeptide–dihydroquinolinone conjugates observed at expected regions and were in accordance with the assumed structures. The IR spectra of mono and dipeptide–dihydroquinolinone conjugates, 1–6, showed characteristic lactam or amide carbonyl peaks around between 1687 and 1623 cm^−1^, whereas the carbamate carbonyl peaks around between 1613 and 1604 cm^−1^.

It was observed that in the mass spectra of all compounds 1–6, there were corresponding molecular ion peaks for assumed structures.

### 3.2. Carbonic anhydrase inhibition

Among the biological activities, human carbonic anhydrase (hCA, EC 4.2.1.1) inhibition has been the subject of several investigations since the discovery of the biological importance of this enzyme in several living organisms^29^. Since many heterocyclic compounds exhibit CA inhibitor properties^6^^,^^30^^,^^31^, we synthesised novel type mono and dipeptide–dihydroquinolinone conjugates to explore their possible carbonic anhydrase enzyme inhibition capacities against human carbonic anhydrase hCA I, II, IX and XII.

In order to explore the inhibitory capacity of all the prepared new mono and dipeptide–dihydroquinolinone conjugates (1–6) have been evaluated by means of a stopped flow CO_2_ hydrase assay against four human (h) CA isoforms (hCA I, hCA II, hCA IX and hCA XII). Inhibition results of the compounds are reported in Table 1, along with those referred to acetazolamide (AAZ), used as standard inhibitor. When the results in Table 1 are analysed, the following structure–activity relationships (SAR) can be obtained.

**Table 1.** Inhibition data of hCA I, hCA II, hCA IX and hCA XII with compounds 1–6 and the standard sulphonamide inhibitor acetazolamide (AAZ) by a stopped flow CO_2_ hydrase assay.

*K_i_* (µM)^a^				
Cmp. no.	hCA I	hCAII	hCA IX	hCA XII
1	>100	>100	86.8	2.0
2	>100	>100	41.2	3.8
3	>100	>100	42.6	8.5
4	>100	>100	65.4	5.7
5	>100	>100	37.7	7.0
6	>100	>100	47.6	8.6
AAZ	0.250	0.012	26.0	0.006

a Mean from three different assays, by a stopped flow technique (errors were in the range of ±5–10% of the reported values).

**Table 2.** Antioxidant activities of the synthesised mono and dipeptide–dihydroquinolinone conjugates.

Comp. no.			Antioxidant activity, %		
	12.5 μg/ml	25 μg/ml	37.5 μg/ml	62.5 μg/ml	125 μg/ml
1	3.8	2.2	2.5	1.6	0.6
2	3.1	2.2	0.0	0.9	0.6
3	0.9	0.3	nd	nd	nd
4	2.5	1.6	0.9	2.2	1.6
5	2.8	1.9	1.9	2.8	2.8
6	1.9	6.3	8.8	16.4	30.8
α-Toc.	62.9	63.4	68.4	72.8	74.0
BHA	61.1	63.0	67.5	71.0	72.4

nd, not detected.

- All compounds were found to be ineffective up to 100 µM concentration against hCA I and hCA II compared to AAZ, which has an inhibition value of 0.250 µM (Table 1).
- All the synthesised mono and dipeptide–dihydroquinolinone conjugates exhibited weak inhibitory properties against hCA IX, with Ki values among the series, ranging from 37.7 to 86.8 µM (Table 1). Among the compounds, it was found that those containing methionine (compounds 2 and 5) had a stronger inhibition capacity with 37.7 and 41.2 K values ​​than others in the series.
- As for the tumour associated isoform CA XII, it revealed to be moderately inhibited by all compounds with *Ki* values among the series, ranging from 2.0 to 8.6 µM (Table 1). However, the results are still lower than the *Ki* value of standard compound AAZ.

### 3.3. Antioxidant testing

#### 3.3.1. DPPH radical scavenging activity

The antioxidant activity of the compounds was determined based on the ability of the antioxidants to act as radical scavengers towards the stable free radical, 1,1-diphenyl-2-picrylhydrazyl (DPPH)^28^.

Monopeptide–dihydroquinolinone derivatives, 1–5, from synthesised compounds generally did not show antioxidant activity compared to standard antioxidant compounds α-tocopherol and BHA. Only the dipeptide, 6, showed some antioxidant activity at a concentration of 125 μg/ml (Table 2).

## 4. Conclusions

Mono and dipeptide–dihydroquinolinone derivatives synthesised within the scope of this study were synthesised by benzotriazole-mediated method with good yields. The synthesised compounds were found to be ineffective up to 100 µM concentration against hCA I and hCA II, whereas it was found to be effective against hCA IX and hCA XII at the studied concentrations. The antioxidant activity of the synthesised compounds were generally found to be ineffective at concentrations of 12.5–125 µg/ml. Only the dipeptide–dihydroquinolinone compound 6 showed an activity at a concentration of 125 µg/ml, close to half the antioxidant values shown by standard antioxidants, α-tocopherol and BHA.
